# Unicystic Mucoepidermoid Carcinoma: A Pitfall for Clinical and Pathologic Diagnosis

**DOI:** 10.1155/2022/2676367

**Published:** 2022-09-13

**Authors:** Xi Wang, Wei Li, Yanrui Feng, Lingchao Liu, Huiying He, Binbin Li

**Affiliations:** ^1^Department of Oral Pathology, Peking University School and Hospital of Stomatology & National Center of Stomatology & National Clinical Research Center for Oral Diseases & National Engineering Research Center of Oral Biomaterials and Digital Medical Devices, Beijing 100081, China; ^2^Research Unit of Precision Pathologic Diagnosis in Tumors of the Oral and Maxillofacial Regions, Chinese Academy of Medical Sciences (2019RU034), China; ^3^Central Laboratory, Peking University School and Hospital of Stomatology & National Center of Stomatology & National Clinical Research Center for Oral Diseases & National Engineering Research Center of Oral Biomaterials and Digital Medical Devices, Beijing 100081, China; ^4^Department of Pathology, School of Basic Medical Sciences, Third Hospital, Peking University Health Science Center, Beijing, China

## Abstract

Unicystic mucoepidermoid carcinoma (UC-MEC) is a rare MEC variant, and its diagnosis is frequently problematic. This study is aimed at summarizing its clinicopathologic characteristics, treatment, and prognosis and proposing key points to avoid missed diagnosis and misdiagnosis in clinical and pathological conditions. This retrospective study included 30 UC-MEC cases, and the clinical findings were collected from the clinical medical records. Radiographic features, histologic behaviors, MAML2 rearrangement by fluorescence in situ hybridization (FISH), and follow-up data were analyzed. Moreover, glandular odontogenic cyst (GOC) and cytadenoma (CA) were used as controls. In the UC-MEC group, 19 patients were female (63%), and 11 were male (37%). The mean patient age was 39.5 (range, 7–72 years). The affected locations included the jaw (8 maxillary, 3 mandibular) and salivary glands (7 parotid, 11 palates, and 1 floor of the mouth). The chief complaint was swelling; the lesions were all cystic, among which 66.7% were well circumscribed and 33.3% poorly defined. Microscopic examination showed two UC-MEC histologic subtypes. Type A presented as a single cyst with mural thickening (8/30, 27%) lined predominantly by epidermoid cells with interspersed intermediate and mucinous cells, and type B (22/30, 73%) showed infiltrative tumor islands in the cystic wall or the surrounding tissue. FISH analysis suggested that approximately 66.7% of UC-MEC harbored a MAML2 rearrangement. During the median follow-up period of 42 months (range, 6–120 months), all type A patients and 68% of type B patients who underwent complete surgical resection lived without relapse. Seven cases with type B cancer that underwent curettage initially had local recurrence. Clinicians and pathologists hardly recognize UC-MEC owing to its cystic architecture. Specific epidermoid, mucous, and intermediate tumor cells, and MAML2 fusion testing, are essential to avoid potential diagnostic pitfalls. Prompting and completing resection surgery with negative margins would have a favorable prognosis.

## 1. Introduction

Mucoepidermoid carcinoma (MEC) is the most common malignancy of the salivary glands, accounting for 30% of salivary gland tumors and 2–4% of jaw tumors [1]. Although the majority of MEC are typical in histology, unicystic MEC (UC-MEC), a rare variant of MEC, poses a great diagnostic and treatment challenge for clinicians and pathologists. To date, only 20 cases of UC-MEC have been reported in the English literature [2–9], which means that more cases are needed to study the clinicopathological features and appropriate treatment choices.

In practice, the large cystic architecture of UC-MEC may be a pitfall in diagnosis. On computed tomography (CT) or contrast-enhanced CT, UC-MEC is commonly characterized by well-circumscribed unilocular radiolucency mimicking benign cysts or tumors. Histologically, an insufficiently deep or bread incisional biopsy, such as fine-needle aspiration biopsy (FNAB), may miss the key diagnostic portion of the tumor because large cysts may sometimes develop in the superficial aspect of the tumor and show only an innocuous part [10, 11].

Recent studies have demonstrated that 33.7–86.6% of MECs harbor CREB-regulated transcriptional coactivator 1- (CRTC1-) mastermind-like gene family-2 (MAML2) translocation [12]. Owing to its high specificity [13], the MAML2 rearrangement is considered a useful ancillary diagnostic tool for MEC diagnosis. However, there are no published reports on MAML2 rearrangement in UC-MEC.

The Armed Forces Institute of Pathology (AFIP) grading criteria for typical MEC (TMEC) generally show a good correlation with clinical outcomes and are considered applicable to UC-MEC [14]. The management of UC-MEC should be tailored to its type, location, and histological grading. However, there are still many disputes regarding their disposal. Some researchers thought the UC-MEC qualifies as a low-grade tumor with indolent clinical behavior and may be more conservatively treated [2]. Generally, low-grade MEC requires surgical treatment only, whereas high-grade MEC requires adjuvant radiation and neck dissection [15]. However, some pathologists have emphasized the importance of radical surgery and adjuvant treatment [8]. The recurrence rate was around 40% for conservative treatment such as enucleation and debridement and 4% for radical treatment such as segmental resection with/without associated adjuvant therapy [16]. In the published cohort, 86% of patients were treated with regional surgical excision. Of these patients, 14% underwent extra lymph node dissection and adjuvant radiotherapy. In order to solve these disputes above, we reported the largest number of 30 UC-MEC cases to date and described the clinicopathologic characteristics, diagnosis, and prognosis of UC-MEC to gain a comprehensive understanding of this unique MEC variant.

## 2. Materials and Methods

### 2.1. Study Population Selection and Follow-Up

The study was approved by the Institutional Review Board of Peking University, School and Hospital of Stomatology (PKUSSIRB-201948111). In the head and neck region, 635 cases of MEC were identified from the surgical pathology database between January 2005 and October 2021. All hematoxylin and eosin- (H&E-) stained slides were reviewed and regraded by two head and neck pathologists (Binbin Li and Huiying He). Inclusion criteria for MEC were proposed when the lesion was showing unicystic architecture and composed of varying proportions of epidermoid cells, mucocytes, and intermediate cells. Patients with metastatic MEC from other primary lesions were excluded. Diagnostic hematoxylin and eosin (H&E) slides, and formalin-embedded (FFPE) blocks were retrieved from the surgical pathology archives.

Glandular odontogenic cyst (GOC) is a developmental odontogenic cyst, while cystadenomas (CA) is a rare benign salivary gland tumor. Both share numerous histopathological features with UC-MEC, such as mucous and eosinophilic cuboidal cells [14, 15]. Thus, six GOC and 19 CA samples were selected as the negative control for MAML2 test [17, 18].

Clinical characteristics were obtained from the electronic medical records, including age at the time of diagnosis, sex, tumor site, symptoms and signs, interval between the initial symptoms and histologic verification, image manifestations, surgical treatment (local recision, complete revision with or without neck lymph node dissection), and adjuvant treatment (radiation therapy, chemotherapy, and/or I^125^ seed implantation). Tumors were staged according to the American Joint Committee on Cancer (Cancer Staging Manual, 8th edition) [19]. Long-term follow-up was available for all patients. Local recurrence and distant metastases were confirmed by clinical examination, imaging, and pathological diagnosis of tissue biopsy. The status at the last follow-up was classified as follows: no evidence of disease, alive with disease, or death from disease. Clinical follow-up information was available for all patients.

### 2.2. Histology

Tumor samples were examined microscopically and graded using the histologic grading system of the AFIP [20]. HE staining, tumor size, growth pattern (well-defined or peripheral growth in nests and islands), mitoses, tumor necrosis, atypical mitoses, nuclear pleomorphism, perineural, bone, vascular, and muscle invasions, lymph node, and distant metastasis were evaluated.

### 2.3. Fluorescent In Situ Hybridization

The paraffin sections were labeled with MAML2 probes for in situ fluorescence hybridization (FISH). A dual-color break probe was used. One end was a 680 kb fragment of the 5′MAML2 gene labeled with ZyGreen, and the other was a 370 kb fragment of the 3′MAML2 gene labeled with ZyOrange (Z-2014-200, Zytovision, Bremerhaven, Germany). One hundred randomly selected nonoverlapping tumor cells were evaluated for the presence of orange, green, or yellow fluorescent signals. A normal situation is defined as the distance between two signal points being smaller than the diameter of one point, and signals separated by a distance greater than a single signal width are regarded as split signals. Rearrangement was considered positive if the split signal was above 15%. Each assay was accompanied by internal and external controls to monitor the correct performance of processed specimens and test reagents.

### 2.4. Statistical Analysis

Statistical analyses were performed using Spearman's correlation and Fishers exact test. Statistical programming was completed in R Studio (version 4.0.0) and GraphPad Prism (version 9.0), using packages “ggplot2.” *P* < 0.05 was considered a statistical significance.

## 3. Results

### 3.1. Clinical Features

Thirty patients with UC-MEC arising from the head and neck were selected. Among them, 19 patients were female (63%), and 11 were male (37%). The age at diagnosis ranged from 7 to 72 years (mean age: 39.5 years old), with approximately 40% of these cases presenting during the third and fourth decades. Nineteen lesions were in the salivary gland; of those, seven were in the parotid gland, eleven in the palatal gland, and one on the floor of the mouth. Approximately 37% lesions were in the jaw, including eight in the maxilla and three in the mandible. Eight maxillary lesions were found in the premolar and molar regions. The mandibular lesion involved an angle extending to the premolar region on the same side. Swelling was the principal finding in all the patients. None of the patients showed any signs of lower lip/facial numbness.

### 3.2. Imaging Features

All lesions behaved as cystic masses on computed tomography (CT). In the UC-MEC group, the average tumor size was 1.7 cm (range 0.7–2.6 cm). In total, 66.7% (20/30) of the lesions were well circumscribed. An unclear definition was present in 33.3% of patients with UC-MEC. In the control group, CT imaging showed circular or oval lesions with well-defined borders in all GOC and CA lesions. The tumor sizes in the control groups ranged from 1.0 to 4.5 cm, with a mean size of 1.8 cm. The average tumor size in the UC-MEC group was smaller than that in the GOC and CA groups; however, the differences were not statistically significant (*P* > 0.05) among them.

### 3.3. Histologic Features

On gross examination of UC-MEC, the masses were generally unencapsulated, and a large cystic space was demonstrated in all cases.

Microscopically, in the UC-MEC group, the cyst was lined by tumor-forming cells (epidermoid cells and clusters of mucus-secreting cells interspersed with intermediate cells), areas of mural thickening, and infiltrative components. Oncocytic proteinaceous material and (pre) calcified bodies were detectable within the cavity. In some cases, papillary projections consisting of epidermoid and mucinous cells are observed inside the inner cystic wall. Notably, proliferative clusters of epithelial cells were observed in the subepithelial area of the fibrocollagenous wall in some of these cases. This cluster was composed of intermediate cells with markedly hyperchromatic nuclei, scattered mucous cells, and polygonal cells showing epidermoid differentiation. Nuclear pleomorphism and anaplasia were minimal; mitosis was rare; and necrosis, perineural invasion, and lymphovascular invasion were undetectable. Detailed clinicopathological data of all UC-MEC are presented in Table 1. Based on the morphological features of UC-MEC, GOC and CA could be considered in the differential diagnosis. The presence of distinct and frequently prevalent clusters of epidermoid and intermediate cells help to rule out CA, respectively. GOC may closely mimic UC-MECs, but epithelial spheres or whorl is a vital character for GOC diagnosis.

**Figure 1:**
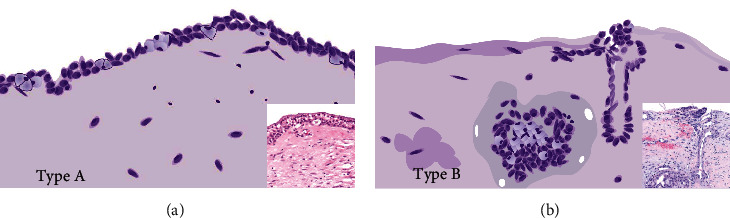
A schematic representation of the types A and B of UC-MEC. Note: UC-MEC: unicystic mucoepidermoid carcinoma.

Taken together, UC-MEC is usually low-grade according to the AFIP grading system, except for one intermediate grade. Cysts occasionally rupture and release mucus into the cyst wall, which may evoke a florid inflammatory and sometimes a granulomatous response or local fibrous repair reaction. Screening of all slides of UC-MEC can be classified into two histologic subtypes. In type A, the tumor was a simple cyst (*n* = 8, 26.7%) with lining epithelium, showing features of an MEC with/without intraluminal tumor nodules. Type B tumors (*n* = 22, 73.3%) contained infiltrative tumor islands in the cyst wall (capsule) or connective tissue. A schematic representation of types A and B cystic MEC is shown in Figure 1.

### 3.4. FISH Results

In the UC-MEC group, 66.7% (20/30) of patients harbored the MAML2 rearrangement, including 62.5% of type A (5/8) and 68.2% (15/22) type B cases.

**Figure 2:**
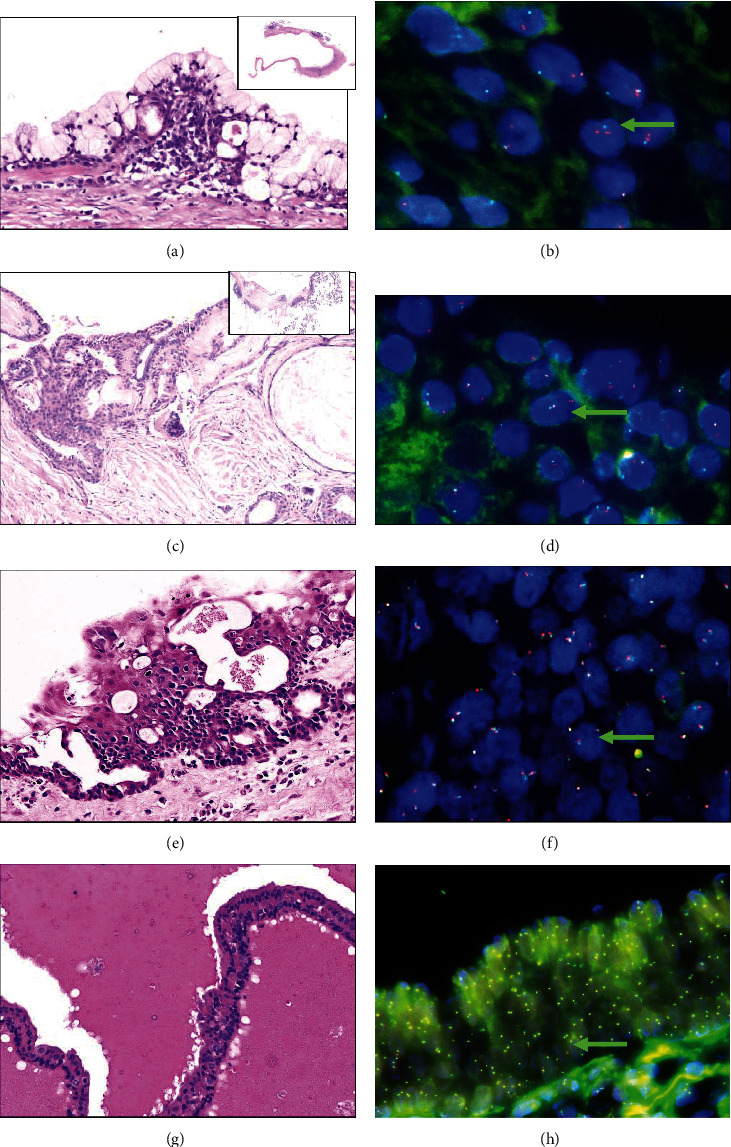
Histological and molecular features of type A, type B MEC, GOC, and CA. (a) Type A UC-MEC (HE ×20); (b) MAML-2 fusion positive in type A UC-MEC (×40), arrowheads indicate MAML2-positive cells; (c) type B UC-MEC (HE ×20); (d) MAML-2 fusion positive in type B UC-MEC (×40), arrowheads indicate MAML2-positive cells; (e) GOC (HE ×20); (f) MAML-2 fusion negative in GOC (×40), arrowheads indicate MAML2-nagetive cells. (g) CA (HE ×40); (h) MAML-2 fusion negative in CA (×40), arrowheads indicate MAML2-nagetive cells.

Six GOC and 19 CA cases were negative for MAML2 gene rearrangements. Representative FISH and histological images are shown in Figures 2(a)–2(h).

### 3.5. Treatment and Follow-Up

Twenty-three patients underwent the extensive resection without recurrence, but 7 cases underwent curettage and recurred (Table 1). Follow-up studies ranging from 6 to 120 months were available for all patients. The overall local recurrence (LR) rate was 23.3% (7/30), and the 5-year LR rate was 16.7% (5/30), of which 2 had an LR beyond five years. The average time to the first LR was 50.8 months (range, 3–108 months). Distal metastases were not observed.

**Figure 3:**
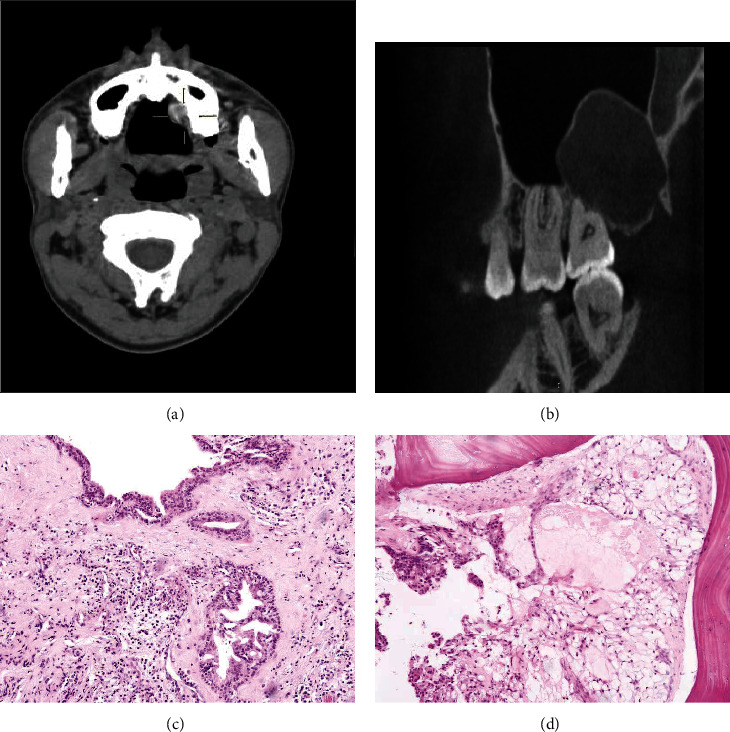
The CT images and histology images of one recurred patient of type B UC-MEC. (a) Contrast-enhanced CT scan of the primary lesion showed that it was cystic and well outlined on the hard palate. (b) 2 years later, CT scan of the recurred lesion showed a well-circumscribed lesion. (c) The proliferative clusters of epithelial cells were observed in the subepithelial area of the fibro-collagenous wall in the primary lesion (HE 10x). (d) The epithelial clusters were invasive into jaw bone in the recurrent lesion (HE 10x).

It is worth mentioning that all relapsed UC-MEC patients were type B and underwent curettage initially; the recurred CT and histology images are shown in Figure 3.

## 4. Discussion

UC-MEC, a MEC variant first described by Raslan [21] in 1998, has been reported as a low-grade tumor with a favorable prognosis. A literature review revealed 20 cases of UC-MEC in the maxillofacial region [2–9]. The average age of these UC-MEC patients was 41.7 (range, 20–80) years. Overall, women (*n* = 16) were more frequently affected than men (*n* = 5). The most frequent primary site was the minor salivary gland (*n* = 18; 9 hard palates, 2 retromolar trigones, 2 mandibles, 2 buccal, 2 soft palates, and 1 mandible), followed by the parotid gland (*n* = 3). Our data confirmed that UC-MEC occurs at an earlier age (mean, 40.3 years) than TMEC (mean, 49 years) [22]. A female predilection in our cohort (63%) was observed, which was consistent with previous reports.

Clinically, the diagnosis of UC-MEC is problematic because of the large number of potential mimics. Swelling was the common cause of hospital visits in patients with UC-MEC, without any malignant indication, and 66.7% of UC-MEC cases were characterized by well-circumscribed unilocular radiolucency with well-defined borders and no signs of malignancy. Therefore, it is difficult for clinicians to distinguish between UC-MEC and benign lesions using only clinical and imaging examinations. In addition, FNAB usually fails to provide sufficient information owing to its cystic nature and insufficient sampling [11]. One recurrent case in our cohort experienced multiple attempts at FNAB over 12 years and failed to identify the malignant nature of the lesion. Incisional biopsy usually consists of only small fragments of the cyst wall, and some nonspecific epithelial lining may not reflect the true nature of the entire lesion. Thus, the negative findings on FNAB do not exclude the possibility of a neoplastic cystic lesion. Therefore, a careful histopathological evaluation of the excised tissue should be performed.

GOC and CA share some histopathological features with UC-MEC, including a cystic architecture lined by epithelium consisting of epidermoid and mucous cells. These morphological similarities make the diagnosis difficult [18]. Apart from the specific features of epithelial plaques in GOC, MAML2 rearrangement tested by FISH or NGS was helpful for differential diagnosis. The most frequent chromosomal translocation in TMEC is t (11; 19) (q21; p12-13), which generates a CRTC1-MAML2 fusion gene. Saade et al. found 77% low- and intermediate-grade MEC harboring MAML2 fusion, and only 23% of high-grade MEC harbored the MAML2 fusion [23]. In our study, MAML2 rearrangement was detected in 55% of UC-MECs, but in none of the GOCs and CAs, which is similar to a previous study [17, 24]. The high specificity of MAML2 rearrangement for UC-MECs points to its utility as a diagnostic adjunct in separating UC-MEC from mucinous cystic lesions in the jaw and salivary glands.

The AFIP grading systems cited by the WHO (2017 vision) have been commonly adopted and proven useful for prognostic purposes. This system considers the extension of the intracystic component, the presence of neural invasion and necrosis, mitotic index, and cellular anaplasia. Based on the features of exclusive intracystic growth, minimal nuclear pleomorphism, mitotic figures, and absence of perineural invasion and necrosis, UC-MEC was assigned to low-grade tumors. Furthermore, according to invasiveness, we subtyped UC-MEC into types A and B, showing distinctive behavioral differences within its own histopathologic spectrum. In type A, none of the patients had postoperative recurrence mimicking in situ carcinoma. In type B, infiltrative tumor islands were recognized as exhibiting a higher risk of recurrence than those in type A. The management of UC-MEC should be tailored to its grade, histological subtype, and location. For both types A and B, surgery aims to obtain complete resection with negative surgical margins.

## 5. Conclusions

We collected and analyzed 30 cases of UC-MEC over a long term and clarified the key factors influencing diagnosis and local recurrence. UC-MEC showed two histologic subtypes. In equivocal cases of UC-MEC, FISH for MAML2 rearrangement helps to resolve a differential diagnostic dilemma. Complete surgical resection is a promising treatment option for UC-MEC. The present unique subtype further emphasizes the intralesional heterogeneity of MEC, which may lead to the diagnostic and treatment confusion.

## Figures and Tables

**Table 1:** Clinicopathological characteristics of patients with UC-MEC.

ID	Subtype	Age	Gender	Location	AFIP grading	FISH result	Treatment	Status
Case1	A	27	Male	Palate gland	LG	-	ER	Live, nr
Case2		29	Male	Palate gland	LG	+	ER	Live, nr
Case3		37	Female	Palate gland	LG	+	ER	Live, nr
Case4		7	Female	Palate gland	LG	+	ER	Live, nr
Case5		39	Female	Palate gland	LG	+	ER	Live,nr
Case6		30	Female	Parotid gland	LG	-	ER	Live, nr
Case7		40	Male	Parotid gland	LG	-	ER	Live,nr
Case8		30	Female	Mandibular	LG	+	ER	Live, nr
Case9	B	67	Female	Palate gland	LG	+	ER	Live, nr
Case10		49	Male	Palate gland	LG	-	ER	Live, nr
Case11		24	Female	Palate gland	LG	-	ER	Live, nr
Case12		52	Male	Palate gland	LG	-	ER	Live, nr
Case13		46	Female	Palate gland	LG	+	EN	Live,re
Case14		69	Male	Palate gland	LG	+	ER	Live,nr
Case15		58	Male	Parotid gland	LG	-	ER	Live, nr
Case16		50	Female	Parotid gland	LG	+	ER	Live, nr
Case17		72	Female	Parotid gland	LG	+	ER	Live,nr
Case18		33	Male	Parotid gland	LG	+	ER	Live,nr
Case19		38	Female	Parotid gland	LG	+	EN	Live,re
Case20		45	Male	Floor of the mouth	LG	+	EN	Live,re
Case21		28	Male	Maxillary	LG	+	ER	Live, nr
Case22		40	Female	Maxillary	LG	-	ER	Live, nr
Case23		49	Female	Maxillary	LG	-	ER	Live, nr
Case24		30	Male	Maxillary	IntG	+	EN	Live, re
Case25		23	Female	Maxillary	LG	+	ER	Live, nr
Case26		30	Female	Maxillary	LG	+	ER	Live, nr
Case27		26	Female	Maxillary	LG	-	EN	Live, re
Case28		30	Female	Maxillary	LG	+	ER	Live, re
Case29		57	Female	Mandibular	LG	+	ER	Live, nr
Case30		30	Female	Mandibular	LG	+	EN	Live, re

Note: UC-MEC: unicystic mucoepidermoid carcinoma; LG: low grade; IntG: intermediate grade; ER: extensive resection; EN: enucleation; Live, nr: live, no recurrence; Live, re: live, recurrence.

## Data Availability

The data used to support the findings of this study are available from the corresponding author upon request.
